# Effects of cyclophosphamide and IL-2 on regulatory CD4^+^ T cell frequency and function in melanoma patients vaccinated with HLA-class I peptides: impact on the antigen-specific T cell response

**DOI:** 10.1007/s00262-013-1397-7

**Published:** 2013-04-16

**Authors:** Chiara Camisaschi, Paola Filipazzi, Marcella Tazzari, Chiara Casati, Valeria Beretta, Lorenzo Pilla, Roberto Patuzzo, Andrea Maurichi, Agata Cova, Michele Maio, Vanna Chiarion-Sileni, Gabrina Tragni, Mario Santinami, Barbara Vergani, Antonello Villa, Emilio Berti, Ludmila Umansky, Philipp Beckhove, Viktor Umansky, Giorgio Parmiani, Licia Rivoltini, Chiara Castelli

**Affiliations:** 1 Unit of Immunotherapy of Human Tumors, Fondazione IRCCS Istituto Nazionale dei Tumori, Via G. Venezian 1, 20133 Milan, Italy; 2 Fondazione IRCCS Istituto Nazionale dei Tumori, 20133 Milan, Italy; 3 Present Address: Unit of Medical Oncology, Department of Medical Oncology, Fondazione IRCCS Istituto Nazionale dei Tumori, 20133 Milan, Italy; 4 Melanoma and Sarcoma Unit, Fondazione IRCCS Istituto Nazionale dei Tumori, 20133 Milan, Italy; 5 Division of Medical Oncology and Immunotherapy, Department of Oncology, University Hospital of Siena, Istituto Toscano Tumori, 53100 Siena, Italy; 6 Cancer Bioimmunotherapy Unit, Department of Medical Oncology, Centro di Riferimento Oncologico, Istituto di Ricovero e Cura a Carattere Scientifico, 33081 Aviano, Italy; 7 Medical Oncology Unit, Istituto Oncologico Veneto-IRCCS, 35128 Padua, Italy; 8 Department of Pathology, Fondazione IRCCS Istituto Nazionale dei Tumori, 20133 Milan, Italy; 9 Consorzio MIA (Microscopy and Image Analysis), University of Milano-Bicocca, 20126 Milan, Italy; 10 University of Milano-Bicocca, Milan, Italy; 11 IRCCS-Fondazione Cà Granda Ospedale Maggiore Policlinico Milano, 20122 Milan, Italy; 12 Department of Translational Immunology, German Cancer Research Center, 69120 Heidelberg, Germany; 13 Skin Cancer Unit, German Cancer Research Center, Heidelberg, Germany; 14 Department of Dermatology, Venereology and Allergology, University Medical Center Mannheim, Ruprecht-Karl University of Heidelberg, 69120 Mannheim, Heidelberg, Germany; 15 Present Address: Unit of Immunobiotherapy of Solid Tumors, San Raffaele Scientific Institute, 20132 Milan, Italy

**Keywords:** Regulatory T cells, Cyclophosphamide, Melanoma, Immunotherapy

## Abstract

The frequency and function of regulatory T cells (Tregs) were studied in stage II–III melanoma patients who were enrolled in a phase II randomized trial of vaccination with HLA-A*0201-modified tumor peptides versus observation. The vaccinated patients received low-dose cyclophosphamide (CTX) and low-dose interleukin-2 (IL-2). Tregs were analyzed in the lymph nodes (LNs) of stage III patients who were undergoing complete lymph node dissection and in peripheral blood mononuclear cells (PBMCs) collected before vaccination and at different time points during the vaccination period. The LNs of the vaccinated patients, which were surgically removed after two rounds of vaccination and one dose of CTX, displayed a low frequency of Tregs and a less immunosuppressive environment compared with those of the untreated patients. The accurate time-course analysis of the PBMCs of patients enrolled in the vaccination arm indicated a limited and transient modulation in the frequencies of Tregs in PBMCs collected after low-dose CTX administration and a strong Treg boost in those PBMCs collected after low-dose IL-2 administration. However, a fraction of the IL-2-boosted Tregs was functionally modulated to a Th-1-like phenotype in the vaccinated patients. Moreover, low-dose IL-2 promoted the concomitant expansion of conventional activated CD4^+^ T cells. Despite the amplification of Tregs, IL-2 administration maintained or further increased the number of antigen-specific CD8^+^ T cells that were induced by vaccination as demonstrated by the ex vivo human leukocyte antigen-multimer staining and IFN-γ ELISpot assays. Our study suggests that the use of CTX as a Treg modulator should be revised in terms of the administration schedule and of patients who may benefit from this drug treatment. Despite the Treg expansion that was observed in this study, low-dose IL-2 is not detrimental to the functional activities of vaccine-primed CD8^+^ T cell effectors when used in the inflammatory environment of vaccination.

**Electronic supplementary material:**

The online version of this article (doi:10.1007/s00262-013-1397-7) contains supplementary material, which is available to authorized users.

## Introduction

Regulatory T cells (Tregs) are essential for the maintenance of self-tolerance and immune homeostasis, and their archetypal feature is the ability to suppress the activation, proliferation, and effector functions of a wide variety of immune cells, mostly in a contact-dependent manner [1]. The relevance of CD4^+^ Tregs in suppressing the anti-tumor immune response is well documented, and numerous studies have reported that the accumulation of Tregs in cancer patients is generally associated with tumor progression, a poor prognosis and the suppression of anti-tumor immunity [2, 3]. The influence of Tregs in down-modulating anti-tumor immunity is an area of intense investigation, and several strategies have been employed to either block or eliminate Tregs [4–6]. In murine models, cyclophosphamide [*N*,*N*-bis (2-chloroethyl)-1, 3, 2-oxazaphosphinan-2 amine 2-oxide, the generic name for Cytoxan (CTX) or Endoxan] acts by limiting the number of circulating Tregs [7, 8]. In human settings, the biological activities of CTX are dose-dependent, and metronomic dosing has been demonstrated to limit Treg expansion in patients with advanced cancer [9–11]. However, there is no consensus on the immunological properties of low-dose CTX, most likely because of the different treatment schedules that have been applied and the heterogeneous methods that have been used to detect Tregs [12–15].

Naturally occurring activated Tregs, ready to exert their suppressive activities, are defined as CD4^+^CD25^hi^Foxp3^+^ T cells [16]. The definition of Tregs using multiparametric FACS analysis according to the CD4/CD25/Foxp3 phenotype allows additional functional markers to be combined and the direct ex vivo profiling of these cells, which is crucial because of the functional heterogeneity and the high lineage plasticity of Tregs [1, 17, 18].

CD25 is the α-chain of the IL-2 receptor, which is constitutively expressed by Tregs; therefore, Tregs are highly responsive to IL-2 and IL-2 receptor triggering delivers essential signals for Treg development, homeostasis and function [19]. The in vivo administration of high-dose IL-2 to cancer patients or patients with immune-mediated disease leads to a strong expansion of circulating Tregs [20, 21].

In this study, we extensively monitored the behavior of Tregs in early-stage melanoma patients who received low-dose CTX and low-dose IL-2 in a phase II randomized trial. Tregs in peripheral blood and in LNs were investigated with a combination of phenotypic and functional markers to understand the plasticity of these cells in the context of tumor-specific immunization. Immune monitoring of vaccine-induced T cell responses was performed to determine the outcomes of Treg modulation in the patient immune responses to the vaccine.

## Materials and methods

### Study design and patient eligibility criteria

A phase II randomized study of HLA-A*0201-positive patients with stage IIB/C–III melanoma was conducted. The study included vaccination and observation arms.

The peptides that were used in this study were synthesized under GMP conditions by Merck Biosciences AG, Clinalfa (Läufelingen, Switzerland) (>95 % purity). The vaccine included the following four modified peptides (Altered Peptide Ligands, APL): HLA-A*0201-restricted (Melan-A/MART-1[27L], gp100[210M], NY-ESO-1[165V], and Survivin[97M]). For each peptide, 250 μg was emulsified in 0.5 ml Montanide-ISA51 (Seppic, Franklin Lakes, NJ, USA) and injected subcutaneously (s.c.). The first two vaccines were injected in the proximity of the LNs to be dissected by surgery: (1) inguinal LNs: in the upper part of the thigh, approximately 5–6 cm from the inguinal ligament and (2) axillary LNs: in the center of the deltoid area. The other vaccinations were injected close to the inguinal and axillary lymph node regions, the different LN stations were rotated, and the dissected station was excluded. In addition, the patients in the vaccination arm received CTX (Endoxan) (300 mg/m^2^ i.v.) and IL-2 (Proleukin) (3 × 10^6^ IU, s.c.). The vaccination schedule and the drug administration schedule are detailed in Online figure 1.

Patients were selected after primary tumor removal and enrolled at the Fondazione IRCCS Istituto Nazionale dei Tumori of Milan, Azienda Ospedaliera Universitaria Senese of Siena and Istituto Oncologico Veneto of Padua. The protocol was conducted in compliance with the Declaration of Helsinki and was approved by the ethics committees of each institution. Written informed consent was obtained from each subject. Patients with histologically confirmed (American Joint Committee on Cancer) stage IIB/C–III resectable melanoma (ECOG score 0–1) and normal hematopoietic, liver and renal function were eligible. Of the 43 patients who were enrolled, 22 were randomized into the vaccination arm and 21 into the observation arm. Samples from 16 patients in each group were available for the Treg analysis after blood collection. Patients in the observation arm received neither vaccination nor IL-2 and CTX. The PBMCs of stage IV melanoma patients who were admitted to the Fondazione IRCCS Istituto Nazionale dei Tumori of Milan and who were not eligible for this vaccination study were collected after informed consent was obtained. The PBMCs were used to determine the baseline frequency of the circulating Tregs, which is reported in Online figure 3.

### Blood and tissue samples

Blood samples were collected from healthy donors and patients. PBMCs were isolated using Ficoll-Paque™ PLUS density gradient centrifugation as previously described [22]. Patients with tumor-positive sentinel nodes underwent complete lymph node dissection (CLND). The LN material, which was left over by the pathologist after the diagnostic procedures, was immediately processed (within an hour) into a single-cell suspension. Using enzymatic digestion or mechanical dissociation, cell suspensions were obtained from each single LN with a known tumor invasion status based on the pathological assessment, and the suspensions were analyzed as separate samples. For patients with tumor-positive nodes among the lymph nodes that were obtained by CLND, both tumor-involved and tumor-free LNs were examined when available.

The sera were prepared by clotting the blood samples with a clot activator and a separating gel (BD Biosciences, San Josè, CA). After incubation at RT for 30 min, the serum was obtained by centrifugation at 1,800×*g* for 10 min and subsequently stored at −80 °C until use.

### T cell analysis

FACS analysis was performed using thawed PBMCs and LN-derived lymphocytes with the following mAbs: APCH7 or APC-conjugated anti-CD4, PE-Cy7-conjugated anti-CD25, FITC-conjugated anti-CD45RA and fluorochrome-conjugated mouse IgG as an isotype control (all of the antibodies were purchased from BD Biosciences). Intracellular staining with PE- or APC-conjugated rat IGg2a Kappa anti-Foxp3 mAbs (eBioscience, San Diego, CA) was performed according to the manufacturer’s instructions. For each single patient, Treg staining was simultaneously performed. In the analysis, activated Tregs were defined as CD4^+^Foxp3^+^CD25^hi^. The CD25^hi^ region was set to include 100 % positive Foxp3 cells. For some experiments, as indicated in the legends of the corresponding figures, an anti-CD45RA mAb was included to allow for the analysis of conventional activated T cells that were gated as CD4^+^CD25^int^Foxp3^int^CD45RA^neg^.

For the intracellular staining of lymphocytes, PBMCs were freshly isolated or activated overnight with anti-CD3/CD28 beads (DynaBeads^®^ CD3/CD28 T cell Expander, Invitrogen Dynal AS, Oslo, Norway) (the PBMC:beads ratio was 25:1) in the presence of 1 μl/ml Golgi Plug (BD Biosciences). The PBMCs were stained for cell surface markers, fixed and permeabilized with Cytofix/Cytoperm buffer (BD Biosciences) and stained with PE-labeled anti-IFN-γ or FITC-labeled anti-Ki67 (BD Biosciences), PE-labeled anti-T-bet (eBioscience) or PE-labeled anti-TGF-β1 (IQ Products, Groningen, The Netherlands). When combined with the anti-Foxp3 antibody, staining was performed using eBioscience Fixation/Permeabilization buffers.

The distribution of CD25- and Foxp3-positive cells was similar in unstimulated and in ex vivo activated CD4^+^ cells. CD4^+^ cells that exhibited the phenotypic traits of activated Tregs, CD25^hi^Foxp3^hi^, and conventional activated T cells with intermediate expression of CD25 and Foxp3 and no expression of the CD45RA were both clearly evident in ex vivo activated PBMCs.

The fluorescence intensity was measured using a Navios™ (Beckman Coulter, Brea, CA) flow cytometer and analyzed using FlowJo^®^ Cytometry Analysis software (Tree Star Inc., Ashland, OR).

### Isolation of Treg

Tregs were purified from PBMCs using immunomagnetic sorting with the human CD4^+^CD25^+^ Regulatory T Cell Isolation Kit according to the manufacturer’s instructions (Miltenyi Biotec, Bergisch Gladbach, Germany). The purity of the isolated cells (>95 %) was determined using surface staining with anti-CD4 and anti-CD25 mAbs.

### Suppression assay

In vitro suppression assays were performed in 96-well round-bottom plates and evaluated using a CFSE proliferation assay as previously described [23]. The proliferation of responder T cells was evaluated after 72 h.

### Bio-Plex assay

Snap-frozen primary tumor samples were mechanically disrupted and were treated with lysis solution (Bio-Rad Laboratories, Inc., Hercules, California, USA). After sonication, the samples were centrifuged at 4,500*g* for 10 min at 4 °C. The protein concentrations in the lysates were determined using the Pierce BCA Protein Assay Kit (Thermo Scientific) and were adjusted to 250 μg/ml using a serum diluent (Bio-Rad). The concentrations of the inflammatory factors in the tissue lysates were measured using multiplex technology (Bio-Rad).

### Cytokine FlowCytomix

The IL-12p70 levels in the patient and healthy donor sera were measured using the FlowCytomix™ Multiple Analyte Detection System (eBioscience) according to the manufacturer’s protocol. The analysis was performed using a BD FACSCalibur^®^ flow cytometer, and the cytokines were quantified using Pro 2.4 FlowCytomix™ software.

### ELISpot

Immunomonitoring was performed using an ex vivo IFN-γ ELISpot assay (1-D1K, Mabtech AB, Nacka, Sweden) after thawed PBMCs were incubated overnight at 37 °C in culture medium as previously described [23]. The data were evaluated using the A.EL.VIS ELISpot Reader (Thema Ricerca, Bologna, IT). The results are presented as the number of APL-reactive cells/2 × 10^5^ CD8^+^ cells.

### Multimer staining

Peptide/HLA-A*0201-multimers were provided by Proimmune (Ltd., Oxford, UK) and were used as previously described [24]. The fluorescence intensity was evaluated using a BD FACSCalibur^®^ flow cytometer and was analyzed using FlowJo^®^ Cytometry Analysis software (Tree Star Inc., Ashland, OR). A phycoerythrin-conjugated HLA-A*0201-negative multimer was used as a control.

### Statistics

Student’s *t* tests and two-tailed Wilcoxon matched pairs tests (confidence interval [Cl] 95 %) were used as indicated in the figure legends. The statistical calculations were performed using Prism5 software (GraphPad Software, La Jolla, CA, USA). A *p* value ≤0.05 was considered statistically significant.

## Results

### Patient treatment

HLA-A*0201-positive stage IIB/C–III melanoma patients were enrolled in this phase II study and were randomized into two different arms: vaccination or observation only. The patients in the vaccination arm received the following four modified peptides (Altered Peptide Ligands, APL): HLA-A*0201-restricted (Melan-A/MART-1[27L], gp100[210M], NY-ESO-1[165V], and Survivin[97M]). The patients in the vaccination arm received low-dose CTX (300 mg/m^2^ i.v.) as a potential Treg-depleting agent 1 week before vaccination and 7 and 11 weeks following the initiation of vaccination. To boost the vaccine-induced immune responses, these patients received three daily injections of low-dose IL-2 (3 × 10^6^ IU) at a late administration time period, i.e., at weeks 13 and 15, according to a strategy that was suggested by Slingluff and collaborators [25]. In the control arm, the patients received no treatment other than surgery. The patient characteristics, treatment schedule, and study design are detailed in the Online table 1 and in the Online figure 1. The clinical outcomes of this study are reported elsewhere [26].

### Lymph nodes of vaccinated patients displayed a less immunosuppressive environment

**Fig. 1:**
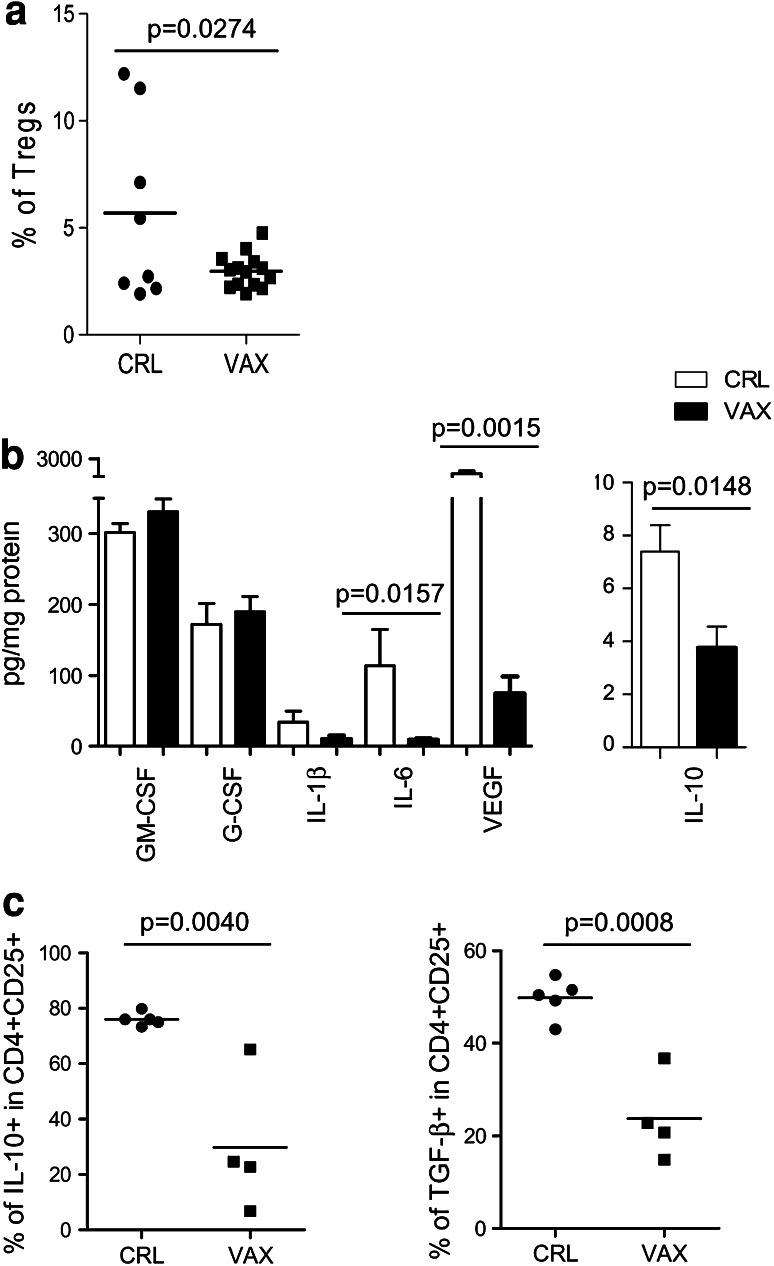
The LN environment of the vaccinated patients was less immunosuppressive than that of the control patients. **a** The lymphocytes that were obtained from patient LNs were stained with anti-CD4, anti-CD25, and anti-Foxp3 mAbs and were assessed by FACS analysis. A comparison between the Treg frequency (CD25^hi^Foxp3^+^ in CD4^+^ cells) in the control patient LNs (CRL, *n* = 8) and in the vaccinated patient LNs (VAX, *n* = 13) was made. **b** The GM-CSF, G-CSF, IL-1β, IL-6, VEGF, and IL-10 levels were detected in the LNs using a Bio-Plex assay and expressed as pg/mg protein (the mean and the SEM) in the control (CRL, *white bars*, *n* = 3) and vaccinated (VAX, *black bars*, *n* = 5) patients. **c** The lymphocytes from the LNs of the control (CRL, *n* = 5) and vaccinated (VAX, *n* = 4) patients were activated ex vivo with anti-CD3/CD28 beads and were stained for the surface expression of CD4 and CD25 and the intracellular expression of IL-10 and TGF-β1. The graphs show the percentages of the IL-10-producing cells (*left panel*) and TGF-β1-producing cells (*right panel*) in the CD4^+^CD25^+^ lymphocytes. The significant *p* values were calculated using Student’s *t* test

Based on a positive sentinel node biopsy, the stage III melanoma patients who were enrolled in the study underwent surgical LN dissection. According to the treatment schedule, the patients in the vaccination arm received one dose of the CTX treatment and two rounds of vaccination before regional LN removal (Online figure 1). The LN material, which was left over by the pathologist after the diagnostic procedures, was immediately processed into a single-cell suspension and assessed by FACS analysis for the presence of CD4^+^CD25^hi^Foxp3^+^activated Tregs. Cell suspensions were obtained from each single LN with a known tumor invasion status according to the pathological assessment, and the suspensions were analyzed as separate samples. The LNs of the vaccinated patients (14 LNs, of which *n* = 4 had macroscopical metastases) were more homogenous according to their Treg content and displayed a low Treg frequency compared with those of the patients who only received surgical treatment (8 LNs, of which *n* = 2 had macroscopical metastases) (% of Tregs mean ± SEM: 2.9 ± 1.1 and 5.6 ± 1.5 in the vaccinated and control LNs, respectively; *p* = 0.0274) (Fig. 1a). In addition, the different Treg distributions in the LNs of the vaccinated and control patients were confirmed by IHC (Online table 2).

To determine whether the CTX and vaccination treatments impacted the immunological milieu of the tumor microenvironment, we investigated the presence of chronic inflammatory mediators in the lysates that were prepared from metastatic LNs, which were surgically removed from the control (*n* = 3) and vaccinated (*n* = 5) patients using a Bio-Plex assay. As shown in Fig. 1b, the majority of the tested inflammatory factors was detected at significantly lower levels in the LNs of the treated patients compared with those of the control patients. Significant reductions in the IL-10 (*p* = 0.0148), IL-6 (*p* = 0.0157), and vascular endothelial growth factor (VEGF) (*p* = 0.0015) levels were evident in the LNs of patients who received CTX and vaccination, which suggests that an amelioration of the local immunosuppressive networks occurred. To confirm these findings, IL-10 and TGF-β1 production by the LN-resident CD4^+^CD25^+^ T cells was evaluated ex vivo upon TCR triggering in 9 additional LNs (5 control LNs, of which 0 had macroscopical metastasis and 4 vaccination LNs, of which 2 had macroscopical metastases). Figure 1c shows that the CD4^+^CD25^+^ cells, which secreted these suppressive cytokines, were decreased in the LNs of the patients who received CTX and vaccination compared with those of the control patients [% of IL-10 in CD4^+^CD25^+^ mean ± SEM: VAX (29.8 ± 12.4) vs. CRL (76.1 ± 1.1); % of TGF-β1 in CD4^+^CD25^+^ mean ± SEM: VAX (23.7 ± 4.6) vs. CRL (49.8 ± 1.9)]. In contrast, similar levels of GM-CSF were detected both in the LNs of the treated patients and in those of the control patients, which suggests that this cytokine was not affected by the CTX/vaccination treatments and supports the in vivo generation of MoDCs in tumor-draining LNs during vaccination.

### CTX- and IL-2-induced modulation of circulating Tregs in vaccinated melanoma patients

The frequency of circulating Tregs was monitored in the peripheral blood of vaccinated and unvaccinated patients by FACS analysis at different time points.

**Fig. 2:**
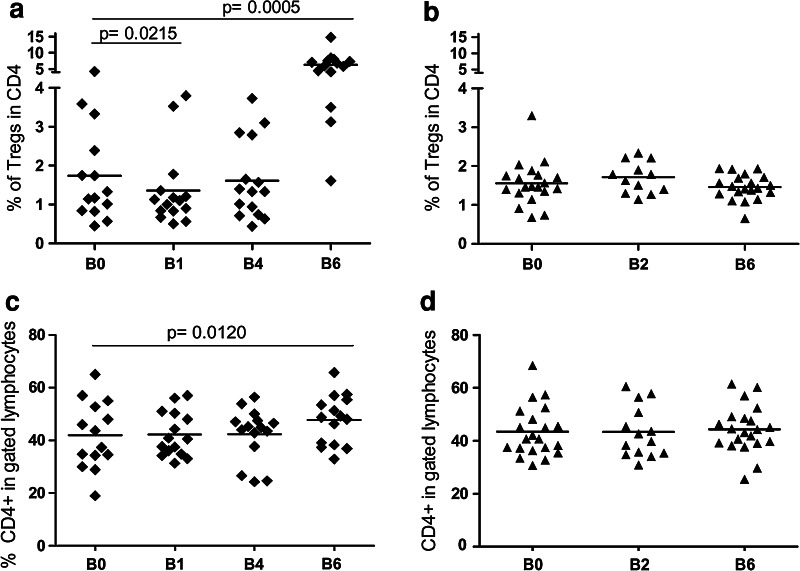
CTX- and IL-2-induced Treg modulation. Patient PBMCs were collected at different time points during the vaccination (**a**, **c**) and observation (**b**, **d**) periods (see the Online figure 1). The PBMCs were stained with anti-CD4, anti-CD25, and anti-Foxp3 mAbs and were analyzed by FACS. The *graphs* show the CD4^+^CD25^hi^Foxp3^+^ Treg frequency (Tregs) in the gated CD4^+^ T subset (**a**, **b**) and the frequency of CD4^+^ cells in the gated lymphocytes (**c**, **d**) (the *p* values were calculated using Wilcoxon statistical tests)

The PBMCs that were obtained from treated patients 4–7 days after CTX administration (B1, Fig. 2a) displayed a slight although statistically significant decrease in the frequency of activated CD4^+^CD25^hi^Foxp3^+^ Tregs compared with the pre-treatment samples (B0, Fig. 2a) (% of Tregs mean ± SEM: B0 (1.7 ± 0.3), B1 (1.3 ± 0.2); *p* = 0.0215). However, the reduction in the frequency of Tregs was transient, and at week 8 (B4, Fig. 2a), the number of Tregs returned to the initial pre-treatment levels.

As expected, the late administration of low-dose IL-2 (3 × 10^6^ IU s.c. for 3 days at week 13 and 15, see Online figure 1) was associated with a slight but significant expansion of the CD4^+^ T cell compartment (Fig. 2c) and caused a robust increase in the number of circulating CD4^+^CD25^hi^Foxp3^+^ Tregs at B6 (i.e., at day 7 after the last IL-2 administration) (% of Tregs mean ± SEM: B0 (1.7 ± 0.3), B6 (6.3 ± 0.8); *p* = 0.0005) (Fig. 2a). The representative time-course analysis results, which are available in Online figure 2, indicate that an increase in the CD4^+^CD25^hi^Foxp3^+^ Treg population occurred at day 7 after IL-2 administration (B6); however, this increase was transitory and was no longer detectable after 15 days (B7). No significant variations in the Treg and CD4 frequencies were detected in the patients who were enrolled in the control arm (Fig. 2b, d). In Tregs, stable *FOXP3* expression is ensured by the demethylation of a conserved region of the *FOXP3* intron 1 [27]. Therefore, methylation-specific quantitative PCR assays (MS-qPCR) can be used as an alternative method to detect Tregs. The down-modulation of the Treg frequency at days 4–7 after CTX administration and the boost of Tregs at B6 (day 112), induced by IL-2, were confirmed by this alternative MS-qPCR assay [28, supplementary data].

### IL-2-expanded circulating Tregs exerted suppressive activity

**Fig. 3:**
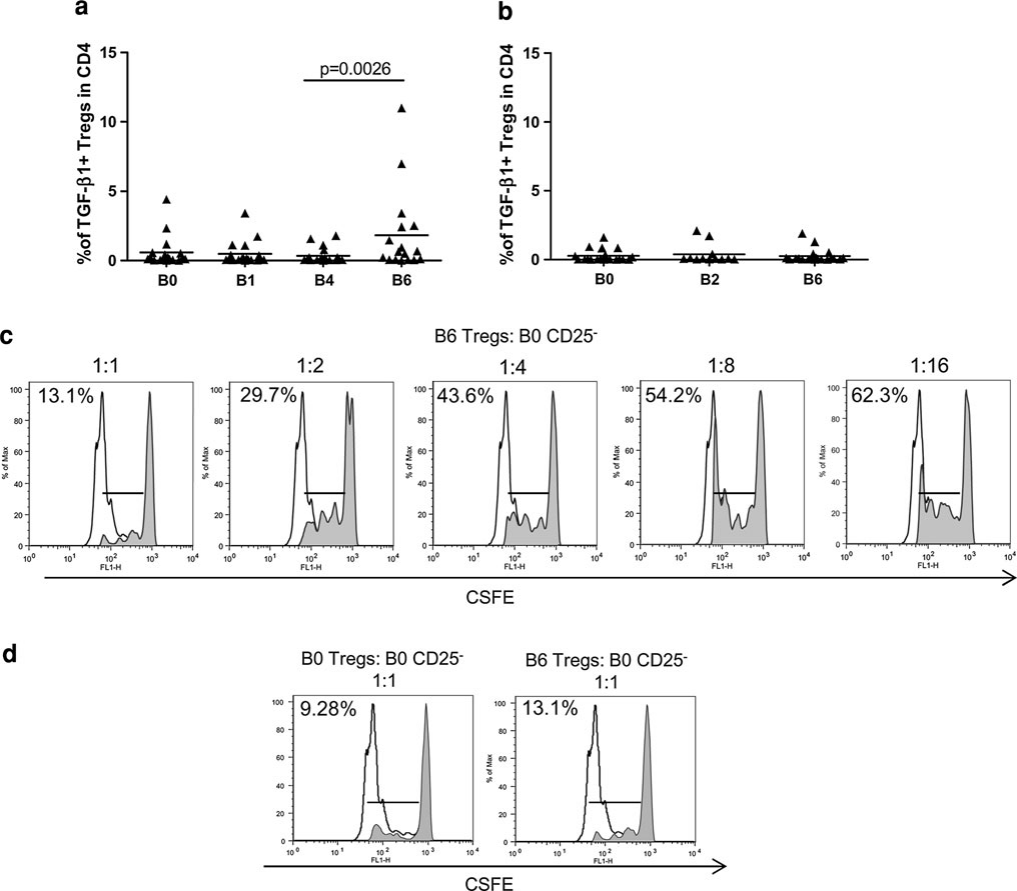
The Tregs expanded by IL-2 exerted suppressive activity. Patient PBMCs were collected at different time points, activated ex vivo with anti-CD3/CD28 beads and stained for Treg markers and TGF-β1. The graphs show the percentages of TGF-β1^+^ Tregs among the CD4^+^ lymphocytes in the vaccinated patients (**a**) and in the control patients (**b**) at different time points (the *p* value was calculated using Wilcoxon statistical tests). **c**, **d** CFSE-labeled CD4^**+**^CD25^**−**^ T cells were stimulated with anti-CD3/CD28 beads and assessed for their proliferative capacity after 72 h in the presence of a decreasing ratio of autologous Tregs (shaded histograms). The unshaded histograms represent unlabeled CD4^+^CD25^−^ T cells. CD4^**+**^CD25^**−**^ responder T cells were isolated from PBMCs at week -1 (B0 CD25^−^). Tregs were purified from the PBMCs of the same patient at week -1 (B0 Tregs) or after IL-2 administration (B6 Tregs). Proliferation was reported as the mean percentage of two technical replicates. The results from one (Pt# 4) out of 3 patients who were studied are shown

The ex vivo functional status of circulating CD4^+^CD25^hi^Foxp3^+^ Tregs was assessed by analyzing TGF-β1 production upon TCR stimulation. As reported in Fig. 3a, a significant boost in the frequency of TGF-β1-producing Tregs was detected in the PBMCs of the vaccinated patients collected 4–7 days after IL-2 administration (B6) [TGF-β1-producing Tregs mean ± SEM: B6 (1.8 ± 0.7) vs. B4 (0.3 ± 0.1), *p* = 0.0026]. No significant variation in the number of TGF-β1-producing Tregs was instead observed in the PBMCs of the patients who were enrolled in the control arm (Fig. 3b). Then, we assessed whether the Tregs in the PBMCs after IL-2 administration could inhibit the proliferation of conventional CD4^+^CD25^−^ T cells. In vitro suppression assays were performed using responder CD4^+^CD25^−^ T cells that were isolated from autologous, pre-vaccine PBMCs (B0, week-1). Figure 3c shows that the Tregs that were isolated 4–7 days after IL-2 administration (B6, week 16) were endowed with the expected, ratio-dependent inhibitory activity. The extent of the suppression that was exerted by post-IL-2 Tregs was similar to that of the pre-treatment Tregs isolated from PBMCs at B0 in the same patient (Fig. 3d).

### An increase in the number of Tregs by IL-2 did not limit the vaccine-induced immunity in melanoma patients

**Fig. 4:**
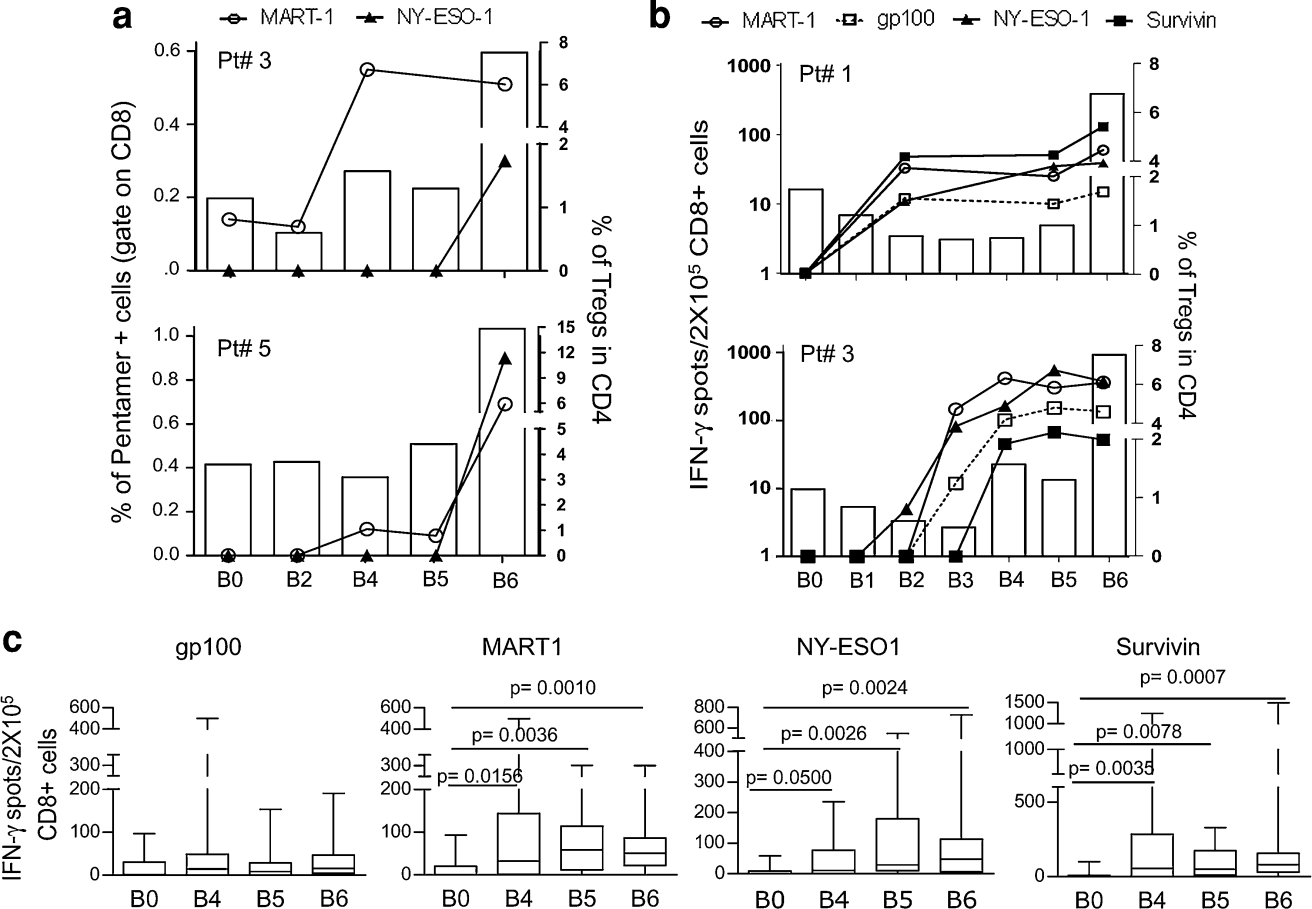
The CD8^+^ T cell response to the vaccine peptides was not affected by Treg expansion. **a** The PBMCs from the vaccinated patients were analyzed ex vivo using HLA-A*0201/peptide multimer staining at different time points during the vaccination period. The percentage of multimer-positive cells were calculated in the CD8^+^ gate (points and connection lines) and reported with the percentage of CD4^+^CD25^hi^Foxp3^+^ Tregs (*white bars*) in the PBMCs from two representative patients (Pt# 3 and Pt# 5). **b** The CD8^+^ T cell response to the vaccine peptides was measured by IFN-γ-ELISpot assay (points and connection lines), and the percentage of CD4^+^CD25^hi^Foxp3^+^ Tregs were measured by FACS analysis (*white bars*). Two representative patients are shown (Pt# 1 and Pt# 3). **c** The CD8^+^ T cells that released IFN-γ in response to the peptides are shown in the graphs at four time points during vaccination: pre-vaccination (B0), after the second vaccination (B4), before IL-2 administration (B5), and after IL-2 administration (B6) (*n* = 16; the *p* value was calculated using Wilcoxon statistical tests. Only the significant *p* values are shown)

The immunological monitoring of the patients who were enrolled in either the vaccine arm or the control arm of the study was performed using HLA-A*0201/peptide multimer (HLA-multimer) staining and IFN-γ ELISpot assays. The robust increase in the Treg frequency at B6, week 16, did not limit the vaccine-induced expansion of peptide-specific CD8^+^ T cells. As reported for two representative patients in Fig. 4a, the pentamer-specific T cells, which were detectable after the 2nd and third vaccinations (B4 and B5), were either maintained or further boosted at B6 after IL-2 administration. More importantly, the antigen-specific CD8^+^ T cells, which were detected at B6 with the concomitant expansion of Tregs, were functionally active and actively released IFN-γ upon antigenic stimulation (Fig. 4b). Ex vivo IFN-γ ELISpot assays indicated that the frequencies of the CD8^+^ T cells that responded to the tumor-associated peptides, which composed the vaccine, were increased after the 2nd vaccination (B2–B5, Fig. 4b). The frequencies of these specific T cells were maintained or further increased after IL-2 administration (B6, Fig. 4b) as confirmed in a larger set of vaccinated patients (Fig. 4c). Further immunological characterization of the vaccine-induced T cell response has been reported elsewhere [26].

### CD4^+^ T cells boosted by IL-2 included T helper type-I-like Foxp3^+^ Tregs and conventional activated T cells

The significant boost in antigen-specific CD8^+^ T cells observed in the peripheral blood concomitantly with the IL-2-mediated increase in Tregs prompted us to further investigate the functional features of the circulating Tregs in the vaccinated patients and better understand the nature of the CD4^+^ T cells that were boosted by the low-dose IL-2 treatment.

**Fig. 5:**
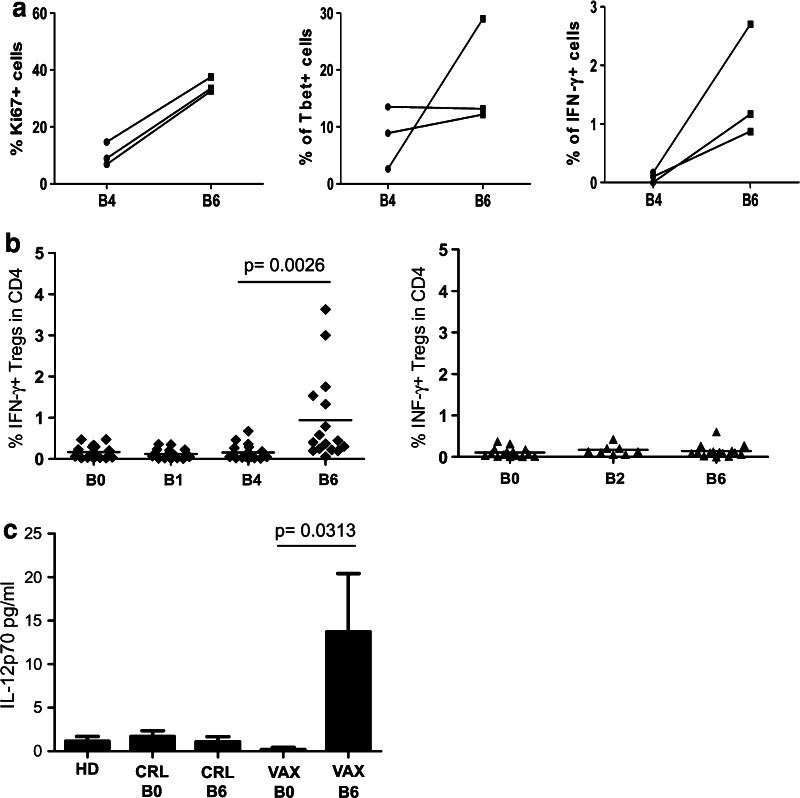
The vaccine-induced Tregs with a Th1-like phenotype. **a** The expression of Ki67, T-bet, and IFN-γ before IL-2 administration (B4) and after IL-2 administration (B6) was evaluated in the Tregs of three vaccinated patients. **b** Patient PBMCs were activated ex vivo with anti-CD3/CD28 beads and were stained for Treg markers and IFN-γ as described in the section Materials and methods. The percentage of IFN-γ^+^ Tregs among the CD4^+^ T cells in the PBMCs of the vaccinated patients (*n* = 16, *left panel*) and the control patients (*n* = 15, *right panel*) are shown (The *p* value was calculated using Wilcoxon statistical tests). **c** The histograms indicate the release of IL-12p70 (pg/ml) in the sera of the healthy donors (HD, *n* = 10), control patients (CRL, *n* = 6) and vaccinated patients (VAX, *n* = 8) pre- (B0) and post-vaccination (B6). [The *p* value was calculated using Wilcoxon statistical tests; the error bars represent the standard error of the mean (SEM)]

In line with the knowledge that activated CD4^+^CD25^hi^Foxp3^+^ Tregs are highly proliferative, we observed that a relatively high percentage of Tregs in both pre- and post-IL-2 PBMC expressed Ki67 (Fig. 5a, left panel). In addition, we observed that activated Tregs that were derived from IL-2-treated PBMCs expressed increased levels of T-bet and accumulated significant amounts of intracellular IFN-γ (Fig. 5a, central and right panels). The presence of IFN-γ-positive Tregs was confirmed after we extended the analysis to all of the vaccinated patients (Fig. 5b, left panel). No IFN-γ-positive Tregs were found in the control patients (Fig. 5b, right panel). Using a gating strategy that selectively considered CD4^+^ T cells with a high expression of CD25 and Foxp3, we excluded conventional activated T cells that expressed CD25 and Foxp3 at intermediate levels [16]. Thus, although INF-γ expression and T-bet positivity are also traits of conventional Th1 cells, our data suggest that a fraction of Tregs, due to their functional plasticity, expressed a T helper type-I-like phenotype in the vaccinated patients. In human autoimmune diseases [29, 30], T helper type-I-like Foxp3^+^ Tregs depend on the availability of the Th1-inducing cytokine IL-12. As shown in Fig. 5c, IL-12p70 was detected in the post-vaccine sera, whereas this cytokine was not observed in the pre-vaccine sera or in the sera from patients in the control arm or from healthy donors [IL-12p70 pg/ml mean ± SEM: VAX-pre B0 (0.2 ± 0.2), VAX-post B6 (13.7 ± 6.7); *p* = 0.0313].

**Fig. 6:**
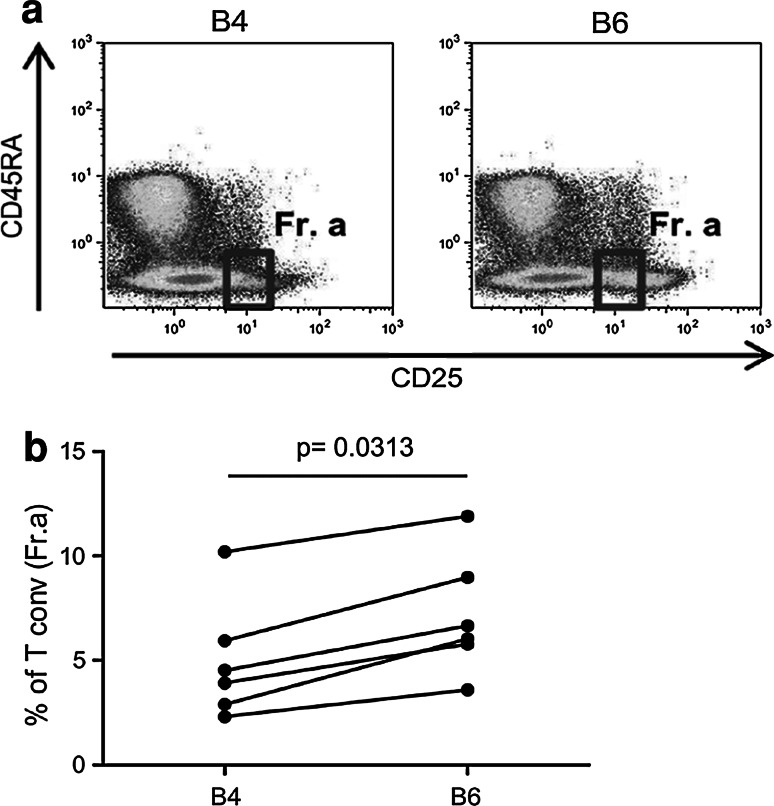
IL-2 administration increased the conventional CD4^+^ T cell frequency. **a** According to Miyara et al. [16], the conventional activated CD4^+^ T cell subset is defined as CD45RA^neg^CD25^low^ (Fr. a), and this subset was detected in the PBMCs before (B4) and after IL-2 administration (B6). The dot plots of a representative patient (Pt# 33) show the FACS gating strategy. **b** The frequency of conventional CD4^+^ T cells (Fr. a) in patient PBMCs before (B4) and after IL-2 administration (B6) is shown (*n* = 6; the *p* value was calculated using Wilcoxon statistical tests)

According to Miyara [16], conventional activated CD4^+^ T cells are defined as CD4^+^CD25^int^Foxp3^int^CD45RA^neg^ cells and they can be discriminated from Tregs. This gating strategy (Fig. 6a, gated as Fr.a) allowed us to further characterize the nature of the CD4^+^ T cells that were boosted by low-dose IL-2. As presented in Fig. 6, IL-2 administration led to the consistent enhancement of conventional activated CD4^+^ T cells in the PBMCs of the treated patients (B4 vs. B6, *p* = 0.0313, Fig. 6b). Taken together, these data confirmed that low-dose IL-2 mediated the expansion of activated conventional CD4^+^ T cells concomitantly with Treg functional plasticity toward a Th1-like phenotype.

## Discussion

The effect of metronomic doses of CTX on circulating Tregs is well documented in advanced cancer patients [7, 8]; however, less and ambiguous data are available on the modulating capacity of this drug when administered at a low-dose in patients with a low tumor burden [12–14]. In addition, data from murine models have indicated that gene and protein expressions are modulated early and transiently by CTX with a maximum effect that is detectable after 24 h of CTX administration; however, no conclusive data have been produced on the kinetics of CTX effects in humans [31]. In our study, we evaluated the effect of low-dose CTX administration on Tregs in the context of a HLA-A*0201-modified tumor peptide vaccine in stage II–III melanoma patients. Our data indicate that CTX slightly decreased the number of circulating Tregs in the vaccinated patients. This reduction, that occurred early after the first CTX injection, was transient despite the subsequent administration of additional CTX doses. In contrast to previous reports, our study included melanoma patients in the early disease stages (stage IIB/C–III) with a baseline frequency of circulating Tregs that was slightly above the values observed in healthy donors and significantly lower than those detected in stage IV melanoma patients (Online figure 3).

In this study, the status of the Tregs in the LNs that were surgically removed from patients with lymph nodal tumor involvement was also assessed. Our data strongly suggest that CTX may be more effective at limiting tumor-induced Tregs at the tumor site rather than the circulating Treg pool. In fact, the frequency of activated Tregs was lower in the LNs of the patients who were treated with CTX compared with that in the LN of control patients. This observation is in agreement with the data that were obtained in pre-clinical models, which demonstrated that CTX selectively depletes the Treg cycling population [32] and Tregs with an effector/memory phenotype and tumor-homing activity [33]. In parallel with this Treg reduction, a low level of immunosuppressive cytokines, such as IL-10, TGF-β1, IL-6, and VEGF, was detected in the LNs of the treated patients compared with the control patients. The LNs were dissected and analyzed in the vaccinated patients after one dose of CTX and after two vaccine injections; therefore, the less immunosuppressive milieu could have resulted from the synergistic interaction between CTX and the stimulating activity of the peptide-based immunization. Because study arms including CTX treatment alone or peptide vaccine treatment alone were not incorporated into the trial design, this hypothesis cannot be further tested. Similarly, the effect of the modulatory activities of CTX and IL-2 on circulating Tregs can be indirectly evaluated by the accurate time-course analysis of the patients who were enrolled in the vaccination arm compared with the patients who only received surgical treatment.

In our study, low-dose CTX demonstrated a limited capacity to modulate circulating Tregs; however, low-dose IL-2 produced a remarkable boost of CD4^+^CD25^hi^Foxp3^+^ Tregs in PBMCs. This enhancement was evident at day 7 after IL-2 administration but was transient and no longer detectable after day 15 (Online figure 2). IL-2-boosted Tregs were immunosuppressive as demonstrated in the in vitro suppressive assay performed on representative cases and by their capacity to produce the immunosuppressive cytokine TGF-β1. TGF-β1 is a key cytokine in Treg-mediated suppression [34–37], and variations in the frequency of TGF-β1-producing Tregs may impact clinical outcomes [38]. Therefore, our data suggest that low-dose IL-2 directly affects Treg homeostasis by inducing the expansion of immunosuppressive Tregs in a similar manner as high-dose IL-2 [20].

Tumor-specific Tregs actively suppress the proliferation of CD4^+^CD25^−^ and CD8^+^ effector T cells, thereby limiting the immune response against cancer and contributing to tumor growth [39, 40]. A recent study found that levels of peripheral Tregs were negatively correlated with the clinical response to adoptive immunotherapy in melanoma patients, and Treg reconstitution depended on the number of IL-2 doses that were administered [41].

In this study, accurate time-course analysis of PBMCs of vaccinated patients indicated that the expansion or the functional activation of antigen-specific CD8^+^ T cells induced by vaccination was maintained or further increased in PBMCs collected after IL-2 administration despite the concomitant boost in Tregs.

Therefore, we investigated the CD4^+^ T cell compartment that was amplified by IL-2 administration in the vaccinated patients. Interestingly, we found that a subset of Tregs in IL-2-treated PBMCs revealed a T helper type 1-like phenotype at the end of the vaccination cycle. This cell subset was recently described in the context of human autoimmune diseases [29, 30] and is IL-12p70 dependent. This cytokine was detectable in the serum of patients post-treatment but not in pre-vaccine sera or in the sera from patients in the control arm. These results confirm the intrinsic plasticity of Tregs and suggest that the pro-inflammatory environment in vaccinated patients can reprogram Tregs into Th-1-like T cells, thus promoting an anti-tumor CD8 response. To our knowledge, these results are the first evidence of Treg functional plasticity occurring in peptide-vaccinated cancer patients. However, the existence of an active interplay between vaccination and the Treg compartment has been previously documented by Romero and colleagues who reported that peptide-based vaccine was able to reduce the frequency of circulating peptide-specific Tregs in melanoma patients [42].

T helper type 1-like Tregs and conventional activated T cells were susceptible to IL-2-mediated expansion. Therefore, as demonstrated in different settings [43], the balance between the different functional T cell subsets that are regulated by IL-2 is a crucial factor that influences the immunological outcomes of vaccinated patients.

Our data emphasize the importance of the functional monitoring of Tregs and suggest that low-dose IL-2 administration in patients receiving peptide-based vaccine, although associated with a boost in circulating Tregs in PBMCs collected after IL-2 administration, was not paralleled by an impairment in the expansion and the effector functions of antigen-specific CD8^+^ T cells induced by vaccination. In contrast, on the basis of our data, the use of CTX requires further study to define the optimal administration schedules and the types of patients who would benefit from this drug.

### Electronic supplementary material

Below is the link to the electronic supplementary material.

Supplementary material 1 (PDF 101 kb)
