# Supplementary material for: Effects of cyclophosphamide and IL-2 on regulatory CD4+ T cell frequency and function in melanoma patients vaccinated with HLA-class I peptides: impact on the antigen-specific T cell response
Source: Cancer Immunol Immunother. 2013 Apr 16;62(5):897–908. doi: 10.1007/s00262-013-1397-7 (PMC3634989; doi:10.1007/s00262-013-1397-7)
Supplement: Supplementary file 1 — Supplementary material 1 (PDF 101 kb) [file 262_2013_1397_MOESM1_ESM.pdf]

“Cancer Immunology, Immunotherapy” Camisaschi C et al.

PATIENT CODE	SEX-AGE	STAGE	ARM	PATIENT CODE	SEX-AGE	STAGE	ARM
**INT01/001**	F61	IIIB	VAX	**INT01/023**	M52	IIIB	OBS
**INT01/002**	F64	IIIB	OBS	**INT01/024**	F56	IIIA	VAX
**INT01/003**	M57	IIC	VAX	**INT01/025**	M30	IIB	OBS
**INT01/004**	M68	IIB	VAX	**INT01/026**	F36	IIIB	OBS
**INT01/005**	F69	IIIA	VAX	**INT01/027**	F41	IIIB	OBS
**INT01/006**	M49	IIIB	VAX	**INT01/028**	F38	IIIB	VAX
**INT01/007**	F28	IIIB	VAX	**INT01/029**	M43	IIIA	OBS
**INT01/008**	M67	IIB	VAX	**INT01/030**	F45	IIB	VAX
**INT01/009**	M44	IIIB	VAX	**INT01/031**	F46	IIIA	VAX
**INT01/010**	M55	IIB	OBS	**INT01/032**	F55	IIB	OBS
**INT01/011**	F38	IIIB	VAX	**INT01/033**	F48	IIIB	VAX
**INT01/012**	M77	IIB	OBS	**INT01/034**	F63	IIB	VAX
**INT01/013**	M68	IIB	VAX	**INT01/035**	M68	IIB	OBS
**INT01/014**	M58	IIC	OBS	**INT01/036**	F36	IIIB	OBS
**INT01/015**	F40	IIB	OBS	**INT01/037**	M38	IIIB	OBS
**INT01/016**	F56	IIB	OBS	**SIE01/001**	M43	IIIC	OBS
**INT01/017**	F49	IIIC	OBS	**SIE01/002**	F51	IIIA	OBS
**INT01/018**	M51	IIIA	OBS	**SIE01/003**	F71	IIIC	VAX
**INT01/019**	F54	IIIB	OBS	**SIE01/004**	F56	IIIA	VAX
**INT01/020**	M60	IIB	VAX	**PDA01/001**	M66	IIB	VAX
**INT01/021**	M56	IIIC	VAX	**PDA01/002**	M46	IIB	VAX
**INT01/022**	M41	IIIB	OBS				

PBMC of patients available for Treg analysis

Online table 1. Patients Characteristics

	Foxp3 EXPRESSION		
SCORE	I	II	III
CRL	0	4	5
VAX	2	6	1

Online table 2. Foxp3 expression in LN tissues

Immunohistochemistry IHC was performed on paraffin embedded LN tissues using anti-Foxp3 mAb. Anti-Foxp3 Ab immunoreactivity was scored as grade I (0%-5%), II (5%-10%) and III (> 10%) according to the number of Foxp3 positive nuclei found in lymphocyte T area; number of positive cases for control patients (CRL, n=9) and vaccinated patients (VAX, n=9) are reported.

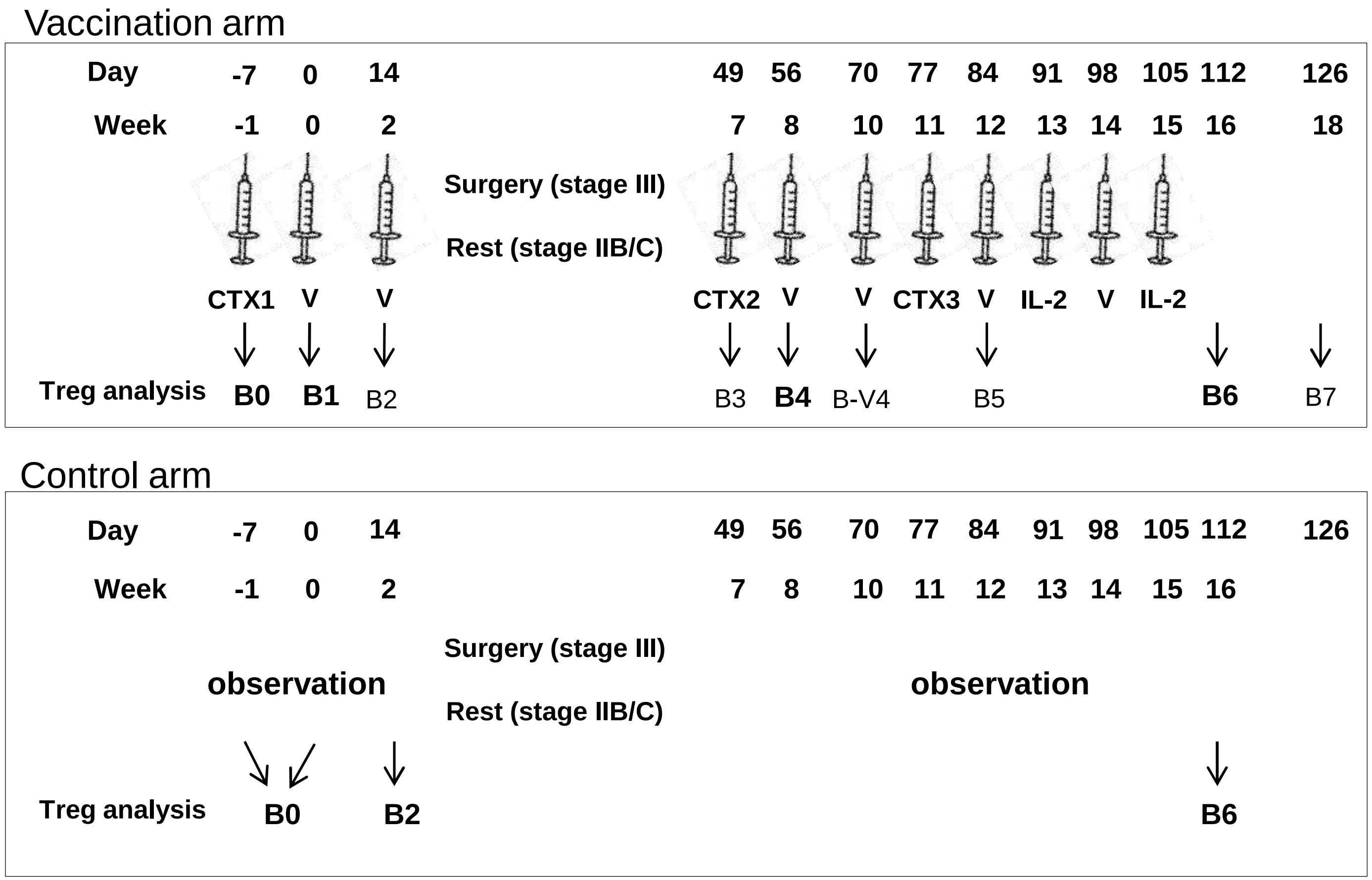

Schematic representation of the vaccination schedule Vaccination arm: V= peptide vaccine comprised of HLA-A*0201-restricted (Melan-A/MART-1[27L], gp100[210M], NY-ESO-1[165V], and Survivin[97M]) 250 µg each emulsified in Montanide ISA-51. CTX= Cyclophosphamide i.v. 300 mg/m^2. IL-2 s.c. 3x10^6 IU/day for 3 days. B= blood samples collected; bold font: samples available for all the patients. Control arm: observation only.

Online figure 1

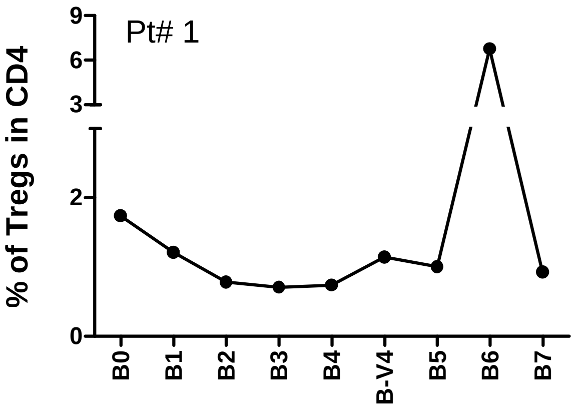

IL-2-mediated Treg increases are transient Graph shows the frequency profile of $CD4^+CD25^{hi}Foxp3^+$ Tregs in gated $CD4^+$ lymphocytes in patient PBMC. A representative case is reported (Pt# 1).

Online figure 2

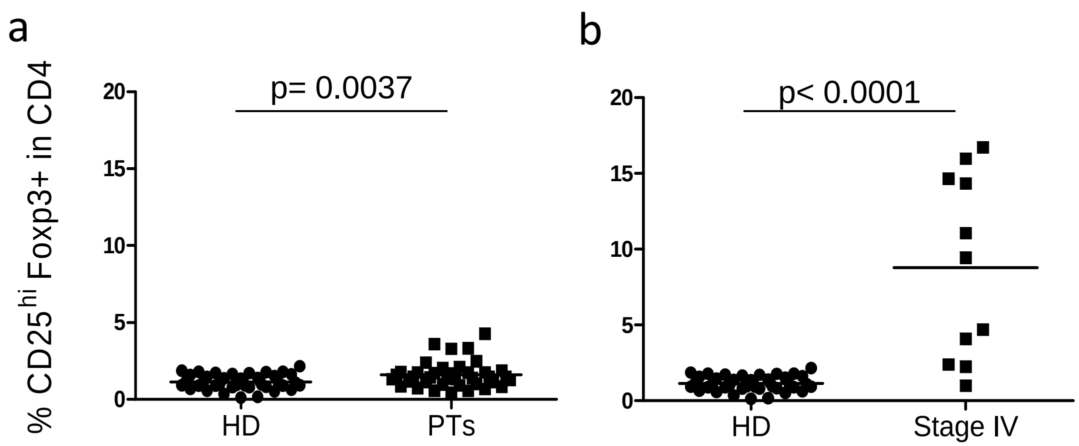

Treg frequency in melanoma patients Difference in the frequencies of Tregs, $CD25^{hi}Foxp3^{+}$ cells in CD4 lymphocytes, between healthy donors (HD, n= 42) and patients enrolled in the study (PTs, n= 32) is reported in panel (a). Panel (b) shows the frequency of Tregs in stage IV melanoma patients with high tumor burden (Stage IV, n= 11) compared to that in healthy donors (HD) (p value calculated with Student's t-Test).

Online figure 3
